# Eating disorder in Down’s Syndrome and GLUT-1 Detection

**DOI:** 10.1192/j.eurpsy.2025.1399

**Published:** 2025-08-26

**Authors:** D. Labunskiy, G. Kukina, S. Kiryukhina, N. Kolmykova, V. Podsevatkin

**Affiliations:** 1 Ogarev Mordovia State University, Saransk, Russian Federation

## Abstract

**Introduction:**

The results of many previous studies report a high prevalence of overweight and obesity in children and adults with Down syndrome (hereinafter DS). According to different authors, the incidence of obesity in them varies between 23 and 70%. The age of onset of overweight formation was 2–3 years.

**Objectives:**

Glucose transporter (abbreviated GLUT) is a large group of membrane proteins responsible for the transport of glucose across the cell membrane. Since glucose is a vital source of energy, these proteins are present in all types of living organisms. GLUT, or SLC2A, is a separate family of glucose transport proteins found in most mammalian cells. Thus, the human genome encodes twelve proteins of the GLUT family. They are transport uniporter proteins.

**Methods:**

We studied PCR of scrapings of the mucous membrane of the inner side of the cheek to search for GLUT1 genes in 31 patients with DS, ьen and women aging from 8 to 39 years. The control group consisted of 67 healthy donors by the same ages and genders.

**Results:**

It was found that the expression of GLUT1 genes was significantly reduced. A decrease in the expression of these genes correlated with an increase in body weight and symptoms of bulimia in patients with Down syndrome.

**Conclusions:**

Eating disorders, namely bulimia, in DS is a serious condition that aggravates the course of the underlying disease. Hereditary factors affect the expression of glucose transporter genes. Taking these circumstances into account would help in developing personalized pharmacotherapy.

**Disclosure of Interest:**

None Declared

